# Characterization of Environmental Dust in the Dammam Area and Mud After-Effects on Bisphenol-A Polycarbonate Sheets

**DOI:** 10.1038/srep24308

**Published:** 2016-04-14

**Authors:** Bekir Sami. Yilbas, Haider Ali, Naseer Al-Aqeeli, Mazen M. Khaled, Syed Said, Numan Abu-Dheir, Necar Merah, Kamal Youcef-Toumi, Kripa K. Varanasi

**Affiliations:** 1 Mechanical Engineering Department and Centre of Excellence in Renewable Energy, King Fahd University of Petroleum & Minerals, Dhahran, Saudi Arabia; 2 Chemistry Department, King Fahd University of Petroleum & Minerals, Dhahran, Saudi Arabia; 3 Mechanical Engineering Department, King Fahd University of Petroleum & Minerals, Dhahran, Saudi Arabia; 4 Mechanical Engineering Department, Massachusetts Institute of Technology, Boston, USA

## Abstract

Owing to recent climate changes, dust storms are increasingly common, particularly in the Middle East region. Dust accumulation and subsequent mud formation on solid surfaces in humid environments typically have adverse effects on surface properties such as optical transmittance, surface texture, and microhardness. This is usually because the mud, which contains alkaline and ionic species, adheres strongly to the surface, often through chemical bonds, and is therefore difficult to remove. In this study, environmental dust and the after-effects of mud formed on a polycarbonate sheet, which is commonly used as a protective glass in photovoltaic cells. Ionic compounds (OH^−^) are shown to significantly affect the optical, mechanical, and textural characteristics of the polycarbonate surface, and to increase the adhesion work required to remove the dry mud from the polycarbonate surface upon drying. Such ability to modify characteristics of the polycarbonate surface could address the dust/mud-related limitations of superhydrophobic surfaces.

Recent climatic changes have produced detrimental effects on the environment, urban life, and industries. For instance, in various geographic regions, climate changes can result in frequent dust storms, subsequently causing dust accumulation on exposed surfaces. Such accumulation can have adverse effects on the sustainability of efficient operations of solar energy-harvesting systems incorporating solar cells and selective surfaces.

The Kingdom of Saudi Arabia is oil rich country, yet it is adorned with plentiful sunlight throughout the year. The renewable energy program has been introduced by King Abdullah City for Atomic and Renewable Energy for the implementation of clean, cost-effective solar energy technologies^1^. One of the aims of the renewable energy program is to have installed sufficient Photovoltaic capacity by 2032 to be able to generate 16GW of power^1^. The environmental effects, such as dust, humidity, and high temperature, influence significantly Photovoltaic device performances^2^. Polycarbonate covers have good mechanical properties and flexibility under thermal expansion load and they are used as protective layers for Photovoltaic panel surfaces. In general, the characteristics of a polycarbonate surface exposed to the environment are degraded by dust accumulation, which in turn degrades the optical, tribological, and thermal properties of the surface. Dust removal requires additional energy and cost to restore the surface to its original state, and is increasingly complicated and challenging in high-humidity environments. Since the city of Dammam is located in the Eastern Province of Saudi Arabia, which is close to the Arabian Gulf, humidity remains high during the periods of spring, summer, and autumn^3^. As dust accumulates, mud forms because of the accumulation of dust particles and the condensation of water vapor on the surface of the dust particles. Dust is composed of soluble particles, such as alkaline metals (e.g., Na, K), and non-soluble compounds such as silica and calcite (CaCO_3_). The soluble compounds alter the base of the solution and increase adhesion of mud onto the surface by forming covalent bonds between the mud and the solid surface. Although adhesion between dry dust particles and the substrate surface is governed by van der Waals forces, the cohesive effect due to the crystallized solution at the interface increases mud adhesion at the surface. Consequently, mud residues that remain at the substrate surface modify the chemical and physical characteristics of the surface including its surface texture, optical and tribological properties, stress levels, and surface hydrophobicity. Such changes can consequently reduce the performance of the solid substrate in a given application. To address this issue, dust accumulation at the surface can be minimized creating surface self-cleaning effects, this procedure fails in the humid air environments because of the excessive interfacial forces generated between the solid surface and the wetted dust particles. Consequently, investigation into mud formation and its effects on solid surfaces are essential.

Bisphenol-A polycarbonate (PC) sheets are used as a protective cover for photovoltaic (PV) panels because of their mechanical flexibility, high fracture toughness, and low density. However, dust accumulation on PC surfaces is problematic and adversely affects the PV efficiency owing to the lowered transmittance of the solar radiation to the active area of the PV panel^4^. In addition, the liquid solution that results from the mud formed in humid environments is chemically active because of the dissolved alkaline materials and alkaline earth metal ions and sediments at the PC surfaceand alter its physical and chemical characteristics. When the solution at the mud–PC interface dries, dissolved minerals crystallize on the surface and the force required to remove the dry dust from the surface increases significantly. The dry mud residues and the crystals of the dried solution degrade the optical transmittance of the PC, thus lowering the PV efficiency.

While airborne dust and its characteristics have been thoroughly examined^5^,^6^,^7^,^8^, studies regarding the effects of environmental dust on surfaces have not been extensively reported^9^. Characterization of atmospheric airborne dust during the wet seasons in East Africa were investigated by Mkoma *et al.*^10^. They demonstrated that common crystal and sea-salt elements, including Na, Mg, Al, Si, Cl, Ca, Ti, Mn, Fe, Sr, NO_3_^−^, and P (and to a lesser extent Cu and Zn) tended to be coarse particles. In addition, the aerosol chemical mass content of the aerosol was determined to consist of 48% organic matter, 44% crustal matter, 4% sea salt, and 2% elemental carbon was observed. A characterization of atmospheric aerosols was also carried out by Maenhaut *et al.*^11^. They showed that most of the Ca was water soluble; the mineral dust Ca was presumably mostly present as calcite, and perhaps also in part as gypsum. In contrast, only half of the K content was water soluble, indicating that it was to a large extent associated with insoluble mineral dust. Patterns of dust retention on urban trees in oasis cities were examined by Baidourela and Zhayimuj^12^. The findings revealed that dust that had accumulated on tree leaves was mainly of local urban origin, and the heavy metal concentrations at different sites varied significantly. The morphology of atmospheric particles in a semi-arid region of India was studied by Mishra *et al.*^13^. They demonstrated that the influence of the dust aspect ratio on dust scattering was significant for dust with a high hematite content. Petruk and Skinner^14^ characterized particles in airborne dust and showed that small particles had low aspect ratios.

Although the relationship between dust accumulation and performance of PV panels has been investigated previously^4^, the after-effects of the mud formed on the PC surface in terms of the surface characteristics have not been addressed. Therefore, in this study, dust characteristics and the after-effects of the mud that forms owing to dust particle accumulation on the surface properties of PC are examined. The surface characteristics investigated include microhardness, surface energy, topology, elemental compositions and compounds, and the molecular states of the surface region. Analytical tools, including optical, electron scanning, and atomic force microscopies, X-ray diffraction, energy-dispersive X-ray spectroscopy, microtribometry, UV transmission spectroscopy, and Fourier transform infrared spectroscopy, were used to characterize the mud after-effects on the PC surface. In addition, the elemental composition of the mud solution was assessed using quadrupole inductively coupled plasma mass spectrometry.

## Experimental

Bisphenol-A PC sheets with size of 6 mm ×6 mm × 3 mm (length, width, and thickness) were used as testing samples. These sheets have excellent optical clarity and high toughness.

### Dust Accumulation and Mud Formation on PC Surfaces

The dust was collected from the solar energy laboratory of research institute at King Fahd University of Petroleum and Minerals, which is located close to the city of Dammam in Saudi Arabia. The dust accumulated on the surface of the protective glass of Photovoltaic panels was collected every two weeks, which was repeated for over the period of twelve months. The dust particles collected were analyzed in terms of weight, size, shape, and elemental composition using the analytical tools. The findings revealed that the dust particles collected over two weeks periods within 12 months have similar characteristics in terms of elemental composition, size distribution, and shape. However, the amount of dust particles accumulated at the surface of the Photovoltaic protective layer 5 g/m^2^ within two weeks; however, it varied within 15% (by weight) over 12 months. This was attributed to wind speed and its direction. Although the wind speed and direction changed over the time, the average wind speed remained about 4 m/s over a year. Dust accumulation and associated mud formation were simulated in laboratory environments that mimicked environmental humid air conditions, in which the air pressure was atmospheric, temperature was kept at 36 °C, and relative humidity was 80%^3^. In the real environment, the condensation of water vapor onto dust particles triggers the formation of mud on the substrate surface. To simulate actual mud formation on the PC surface due to the condensation of water vapor on the accumulated dust particles, the following experiment was conducted. A 300-μm-thick layer of dust particles, which were collected from the local environment, was formed on the PC surfaces. Mud formation on the PC surfaces from the dust particles was then allowed to proceed in a humidity chamber, which simulated humid air conditions. Initial condensation tests were performed in local humid air for a period of 4 hours to estimate the amount of condensate that accumulated prior to dust formation. This step resulted in the natural formation of mud on the PC surface in the chamber. The PC samples with the mud were kept in local ambient air for 3 days to dry. The adhesion work of the sample surfaces with the dry mud was measured. Then, the dry mud was removed from the surface using a desalinated pressurized water jet 2 mm diameter at a velocity of 2 m/s. The cleaning process was undertaken for 20 min for each sample surface tested.

### Characterization

The PC surfaces were characterized using scanning electron microscopy (SEM) and X-ray diffraction (XRD). A JEOL 6460 electron microscope was used for the SEM analysis, and a Bruker D8 Advanced diffractometer with Cu Kα radiation was used for the XRD analysis. The typical settings for the XRD analysis were 40 kV, 30 mA, and scanning angle (2*θ*) of 20–80°. An Atomic Force Microscope (AFM) was used in contact mode, and a tip made of silicon nitride probes (*r* = 20–60 nm) with a manufacturer-specified force constant (*k*) of 0.12 N/m was used.

A microphotonics digital microhardness tester (MP-100TC) was used to measure the microhardness of the surface of the nitride layer. The standard method for Vickers indentation hardness was used (ASTM C1327-99). The microhardness of the PC sheet surface was measured before and after the dry mud was removed. The measurements were repeated five times at each tested location to ensure consistency of the results.

A linear microtribometer (MCTX-S/N 01-04300) was used to measure the tangential and frictional forces to estimate the adhesion work. A contact load of 0.03 N and an end load of 5 N were used. The scanning speed was 5 mm/min and the loading rate was 5 N/s. The total length used during the tests was 1 mm.

A Nicolet Nexus 670 FT-IR spectrometer (Thermo Electron Corporation) was used to measure the Fourier transform infrared (FT-IR) responses of the surface.

Wetting experiments were performed to determine the surface energy of the dry mud using a Kyowa (DM 501) contact angle goniometer and a static sessile drop method. The droplet volume was controlled using an automatic dispensing system with a volume step resolution of 0.1 μL. Still images were captured, and the contact angle measurements were performed 1 s after the liquid droplet was deposited onto the surface. The fluids used for the contact angle measurements were water and glycerol.

The optical transmittance was measured using a UV spectrometer (Jenway 67 Series Spectrophotometer). Fourier transform infrared spectroscopy (Bruker VERTEX70) was performed to examine the infrared spectrum of the absorption of the PC samples.

To examine variations in the pH and elemental composition, the dust particles were mixed with desalinated water and the liquid solution that was extracted from the dust was analyzed using a quadrupole inductively coupled plasma mass spectrometer (Thermo Scientific, XSeries 2).

## Results and Discussion

Characteristics of the environmental dust and the after-effects of mud formed from the dust particles on PC surfaces were examined. Analytical tools were used to assess the mud after-effects on the surface properties of the PC surface including microhardness, mud adhesion, surface energy, topology, elemental composition and compounds, and molecular states of the surface region.

### Analysis of Dust Particles

**Figure 1:**
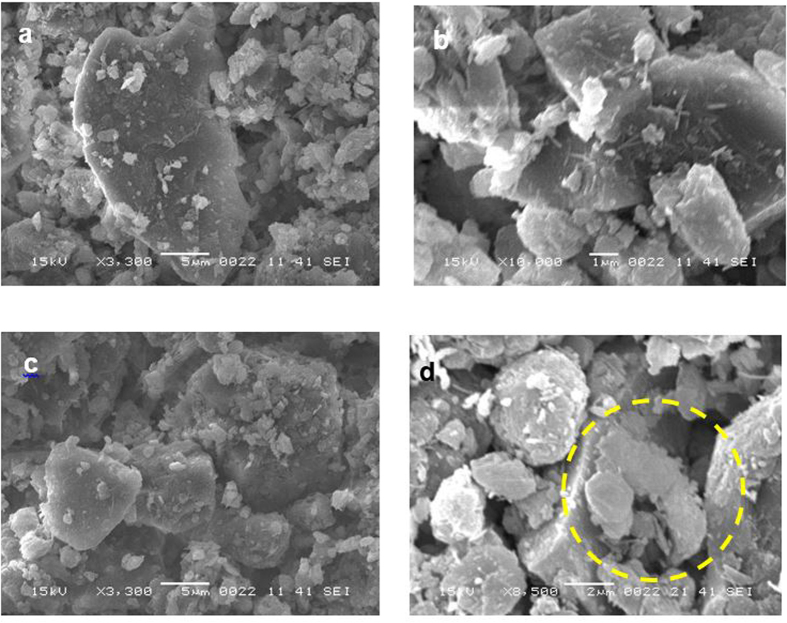
SEM images of the dust particles: (**a**) small particles attached to large particles; (**b**) various sizes of small particles (**c**) mixed sizes of particles and (**d**) flake-like particles shown within the circle.

Figure 1 shows SEM images of the dust particles accumulated on a PV protective glass test surface in Dammam, Saudi Arabia. The size of the dust particles varied from the nanometer range to 30 μm, exhibiting an average size of 1.2 μm. Some of the smaller dust particles in the sub-micrometer range were attached to the surfaces of larger dust particles (Fig. 1a–c). The bright areas typically observed for the smaller particles are indicative of the occurrence of electron charging in the SEM chamber during imaging; this showed that the small particles were charged, which created forces for attachment to the surfaces of the large particles. In general, the dust particles exhibited various shapes with round corners, sharp edges, or flake-like structures.

The geometric features of dust particles can be classified by the shape factor, 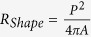, where *P* is the perimeter of the dust particle, and the aspect ratio, 
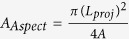
, where *A* is the cross-sectional area, and *L*_proj_ is the longest projection length of the dust particle. The aspect ratio is related to the approximate particle roundness and represents the ratio of the major-to-minor axes of an ellipsoid that is best fit to the particle. The shape factor is the inverse of the particle circularity, which is associated with the complexity of the particle (i.e., a shape factor of unity corresponds to a perfect circle). The particle diameter and area can be obtained from the measurements, where the diameter of a circle with an equivalent area is considered for circular dust particles, and an ellipse model is used when the longest projection is assumed to be the major axis. The particle cross-sectional area is preserved for non-circular dust particles. The relationship between the particle size and the aspect ratio or the shape factor is not simple. An inverse relationship is observed between the particle size and the aspect ratio, whereas a direct relationship is observed between the particle size and the shape factor. In this case, the particle aspect ratio reduces as the shape factor increases with increasing particle sizes. The cross-sectional area for a typical dust particle of size 1.8 μm is in the order of 2.5 μm^2^ and it gives rise to the shape factor of 1.05. However, the cross-sectional area of a typical dust particle of size 15 μm is in the order of 175 μm^2^ and the corresponding shape factor is about 3.18. The shape factor approached unity for the small particles (≤2 μm), whereas for the large particles (≥10 μm), the median shape factor approached 3.

Table 1 shows the elemental composition of the dust particles. In general, Si, Ca, Mg, Na, K, S, O, and Fe were the most common elements detected in the dust particles regardless of the size; however, the concentrations of Na, K, Ca, and O were found to increase, and chlorine was also present in the small particles (≤2 μm). The changes in the elemental composition of the small particles (≤2 μm) can be attributed to the prolonged time the particles spend in the atmosphere and the longer time periods of the particles interaction with solar radiation when compared with the larger particles. Therefore, the prolonged exposure of small-sized particles to the atmosphere allows for the attachment of ionic compounds in regions near the sea.

The EDS data showed that the elemental contents of the particles were non-uniformly distributed regardless of the particle geometrical features, which may be attributed to the geological distribution of the local desert. The concentration of chlorine varied among the different dust particles, and the EDS data were not consistent with the molar ratio of NaCl shown in Table 1. This discrepancy suggests that the dust particles do not contain salt crystals, but rather dissolved chlorinein the compounds. Additionally, the high Si content measured in the flake-like particles (Fig. 1d) indicates that Si was primarily present as silica particles.

**Figure 2:**
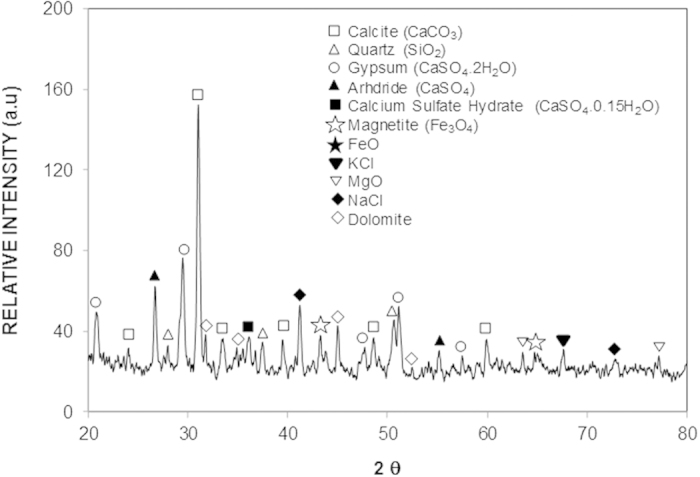
X-ray diffractogram of the dust particles.

Figure 2 shows the X-ray diffractogram of the dust particles and reveals the presence of K, Na, Ca, S, Cl, and Fe peaks. The iron peak coincided with the aluminum and silicon peaks, and the sodium and potassium peaks are likely associated with sea salt because the dust particles were collected from a region near the Arabian Gulf. The presence of sulfur may be related to the presence of calcium, such as anhydrite or gypsum components (CaSO_4_), in the dust particles. Iron is likely associated with the clay-aggregated hematite (Fe_2_O_3_).

### Analysis of the Mud Formed from the Dust Particles

**Figure 3:**
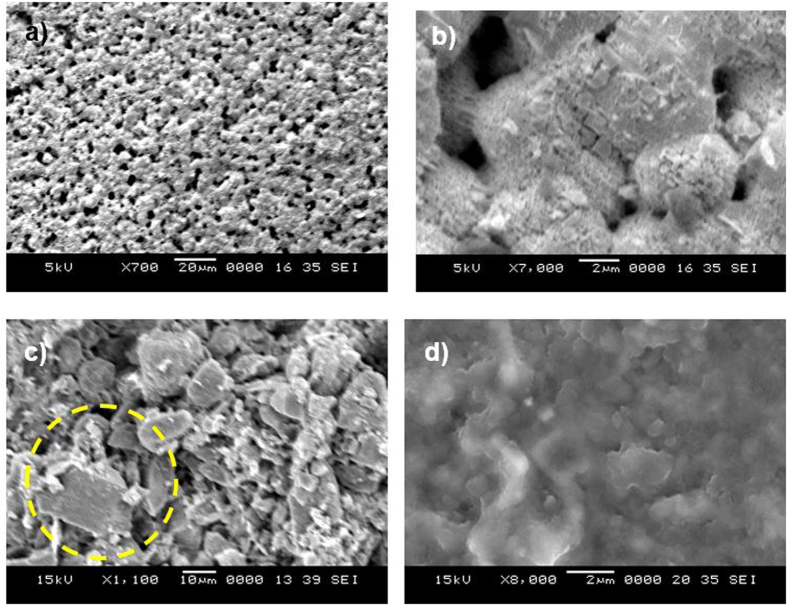
SEM images of the surface of the mud formed from the dust particles: (**a**) fine particles; (**b**) voids between the dust particles; (**c**) large particles and surrounding voids as indicated by the circle; and (**d**) dried mud solution at the mud surface.

**Figure 4:**
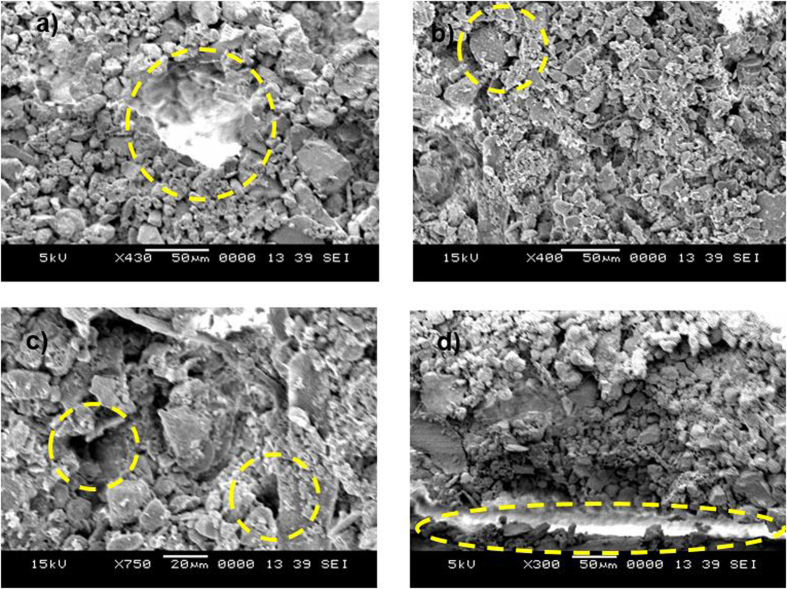
SEM images of the dry mud cross-section: (**a**) small dust particles forming a dense structure and dried solution trapped in the cavity as indicated by the circle; (**b**) large particles as indicated by the circle; (**c**) voids in the dry mud as indicated by the circles, and (**d**) dried mud solution at the interface as indicated by the ellipse.

Figures 3 and 4 show the SEM images of the top surface and cross-section of the dry mud formed on the PC surface, respectively. The dry mud surface was composed of closely packed dust particles (Fig. 4a), micro voids (Fig. 4b), and adhered large dust particles (Fig. 4c). Furthermore, the morphology of the dry mud surface exhibited an irregular topology with an average surface roughness on the order of 2.6 μm. Close examination of the SEM image in Fig. 4d revealed the presence of locally scattered white dense regions on the surface, which is likely related to the dried mud solution that is primarily composed of Na, K, and Ca (Table 2). The alkaline materials (e.g., Na and K) and the alkaline earth metallic compounds dissolved in the water, thus creating a mud solution. Some of the mud solution sediments at the mud–PC interface were obtained upon gravitational settling; however, a small amount remained at the surface where the dust particles were small, and adhered strongly and formed a white residue after drying.

**Figure 5:**
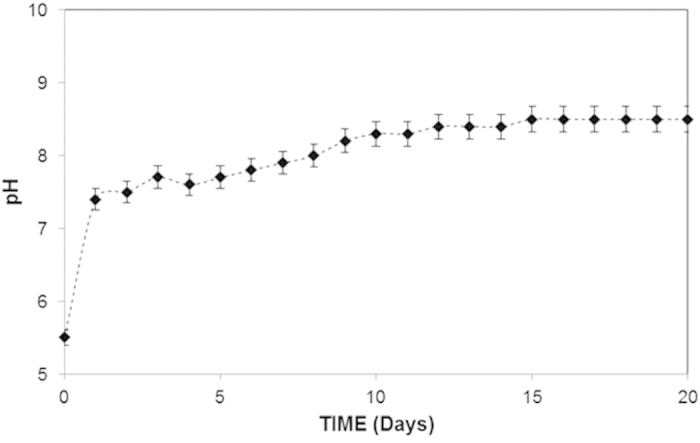
Variation in the pH of the liquid solution consisting of dust particles and water as a function of time.

To determine the pH of the mud solution, the dust particles were mixed with ultra-clean desalinated water at a ratio of 1:4 and placed in an ultrasonic shaker for fifteen minutes. The pH of the mud solution was recorded over a given time period. The temporal variations in the mud solution pH are shown in Fig. 5. As observed, the pH increased with time, and the solution remained basic at pH = 8.4, which is associated with the presence of OH^−^ ions in the solution. In this case, the dissolution of the alkaline and alkaline earth metallic compounds in the water is responsible for the formation of OH^−^ ions. The data obtained from the quadrupole inductively coupled plasma mass spectrometry analysis revealed that the mud solution contained alkaline and alkaline earth metals. Consequently, the sediment mud solution contained alkaline and alkaline earth metals after drying, as evidenced in the EDS data (Table 2).

The surface microhardness data, shown in Table 3, were obtained at different locations on the dry mud surface. The microhardness increased in regions where the small dust particles were in close proximity. This result can be attributed to the strong cohesive forces among the small particles and the presence of the dry solution in this region, which acts as a binding agent among the particles. In contrast, the microhardness was lower in regions containing large particles, which is related to the weak bonding among the large particles due to the voids present among them. The undissolved oddly shaped, large dust particles were responsible for the nearby fine void formations in the dry mud; however, these voids appeared to be randomly scattered on the mud surface. The microhardness values of individual dust particles that were ≥10 μm in size were also measured. The findings showed that the microhardness values varied significantly within the range of 6.7–26.3 HV. In the mud cross-section images (Fig. 4a–d), porous structures were found to cover a large area of the cross-section surface that were likely due to the naturally formed dust layer from the irregularly shaped dust particles. It should be noted that mechanical compaction was not applied to the dust layer on the PC surface prior to mud formation. The liquid solution flowed within the porous structures and hardened at the interface, forming a thin layer between the mud and the PC surface. However, some of the liquid solution remained in the cavities formed between the undissolved irregularly shaped dust particles in the mud. The film formed from the dried solution at the interface contained alkaline elements (e.g., Na, K), alkaline earth metals (e.g., Ca), and Fe, Mg, and Cl, as also shown in the EDS data in Table 2.

#### Analysis of Mud Residues

**Figure 6:**
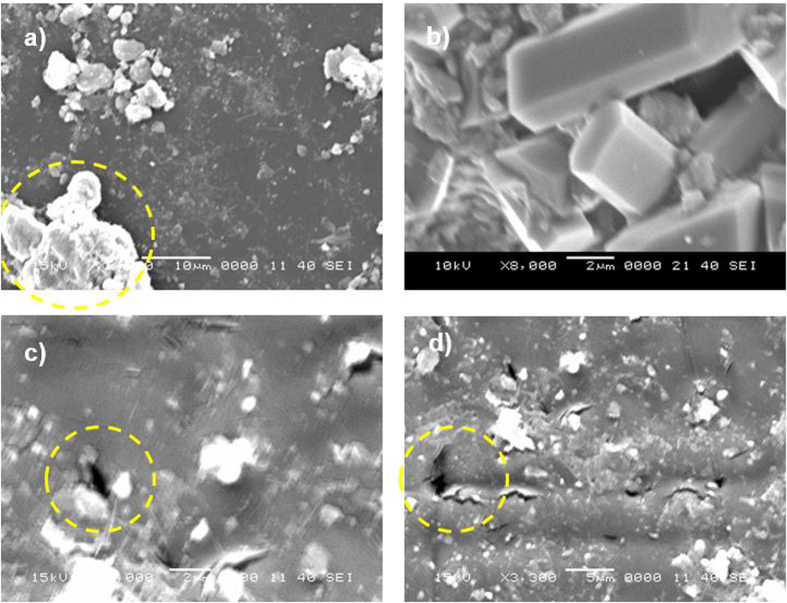
Mud residues and cavities formed at the PC surface after mud removal: (**a**) large and small mud residues—the large residues are indicated by the circle; (**b**) dried solution crystals; and (**c,d**) cavities of various sizes formed at the surface.

**Figure 7:**
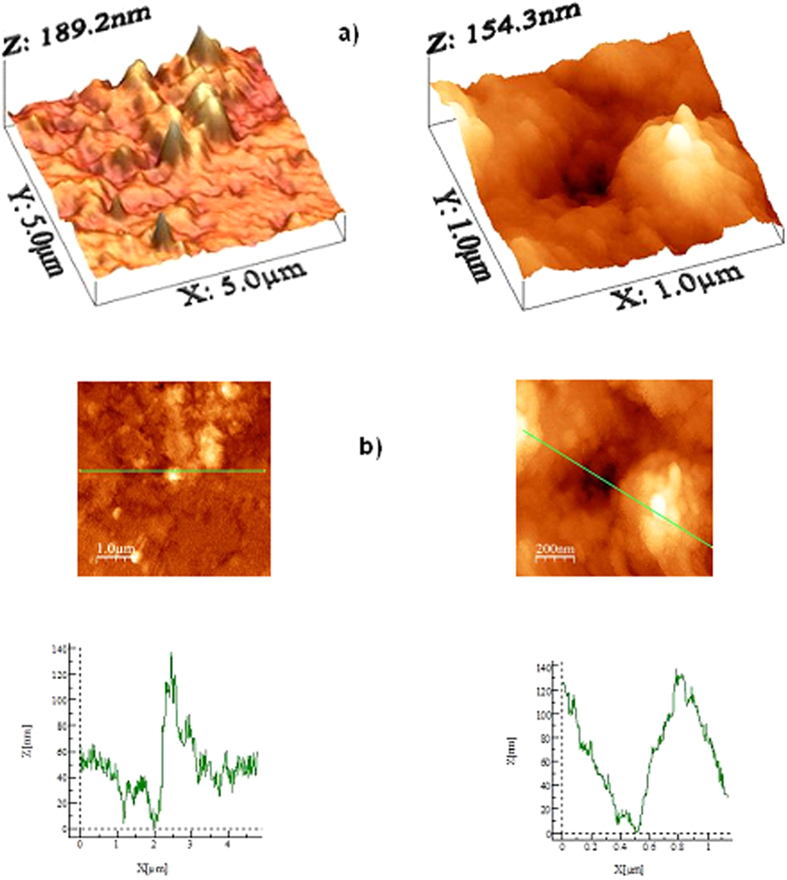
AFM images of the PC surface after mud removal: (**a**) mud residues and the cavity formed in the region near the mud residue and (**b**) line scans of the mud residues and associated profiles of the mud residue and the cavity.

To assess the mud that remained on the surface, the PC surface interface with the dry mud was cleaned using a jet of water. A pressurized water jet that was 2 mm in diameter was sprayed at a velocity of 2 m/s and directed normally to the mud surface during the cleaning process for 20 min. After drying the surface naturally in an air, the PC surface was examined. Figure 6a–d shows SEM images of the PC surface after mud removal. A few locally scattered mud residues were observed on the PC surface (Fig. 6a). Additionally, crystal structures were found on the surface (Fig. 6b). Mud residues were also observed in the AFM micrographs in Fig. 7.

**Figure 8:**
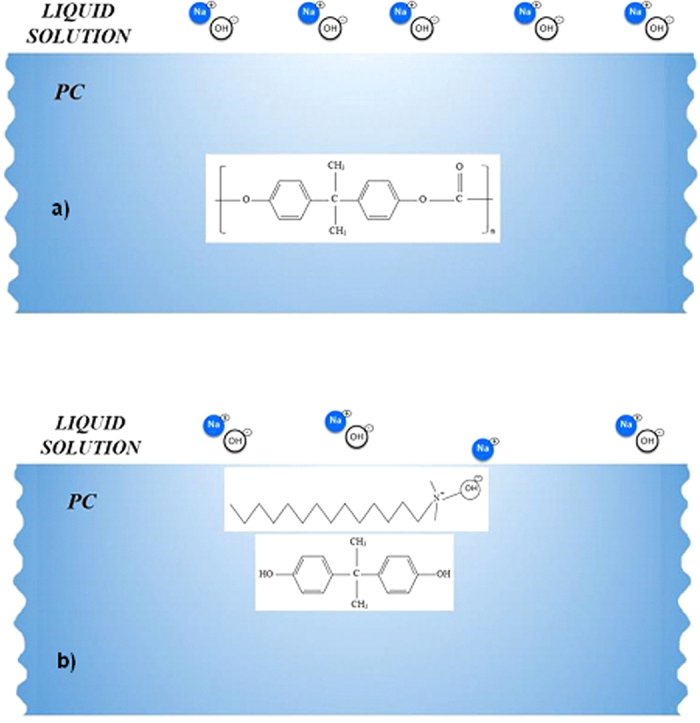
Schematic of NaOH attack of the PC surface: (**a**) the PC surface and the liquid solution and (**b**) PC surface and OH^−^ attachment.

The presence of mud residues after cleaning with pressurized water indicates the presence of strong adhesion between the dry mud and the PC surface. Owing to the mud solution sediment at the interface of the mud and PC, which contains alkaline hydroxides (e.g., NaOH and KOH) due to the high pH (8.4), nucleophile (OH^−^) attacks take place at the PC surface depending on the local concentration of NaOH and KOH in the mud solution. Thus, micromolecules with carbon bonded to three oxygen molecules of the PC (i.e., a carbonate link) at the surface are attacked by OH^−^ due to differences in electronegativity. An anion returns to the aqueous phase, and the reaction continues until complete depolymerization of the PC at the surface. A schematic view of this process is shown in Fig. 8, in which NaOH attack of the PC surface (Fig. 8a) and OH^−^ attachment on PC surface (Fig. 8b) are demonstrated. Similar scenarios apply to KOH. The degradation of carbonate groups leads to a series of reactions due to the elimination of CO_2_ and CO in the absence of free radical reactions (i.e., first-order reaction^15^). Consequently, local degradation creates small cavities at the surface, particularly in the region near the mud residues (Fig. 7a) where the mud solution sediments locally at the surface. The dried mud solution at the interface of the dry mud and the PC surface increases adhesion between the mud residues and the PC surface despite the use of the high-pressure water jet during surface cleaning. Furthermore, the presence of Cl in the dried mud solution at the dry mud–PC interface (Table 2) suggests that the simultaneous presence of bisphenate anions and chlorine could lead to chlorination of the aromatic rings with the formation of polychlorinated bisphenates. However, under basic conditions (e.g., pH = 8.4), polychlorinated bisphenates are known to oxidize to form radicals^16^, in which quinones form depending on the oxidative process that occurs^17^. This process contributes to the degradation of the PC surface, thereby enhancing the formation of small cavities on the PC surface (Figs 6c and 7a).

#### Analysis of As-received PC Sheet and PC Sheet Following Mud Removal

**Figure 9:**
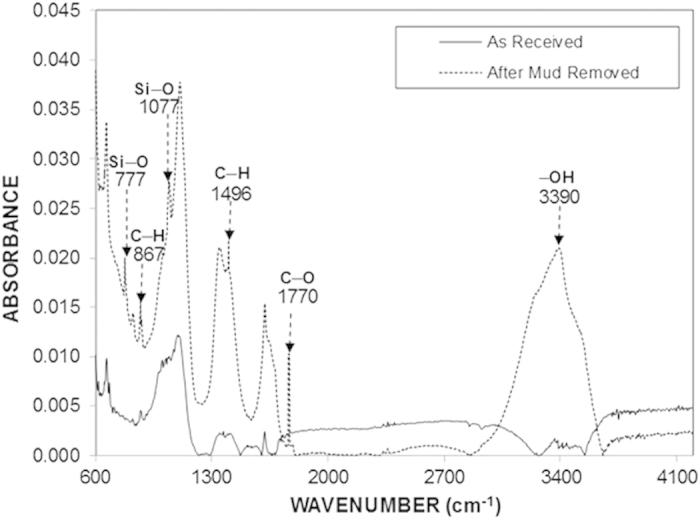
FTIR spectra of the as-received PC sheet and the PC sheet following mud removal.

Figure 9 shows the FTIR spectroscopy data of the as-received PC sheet and PC sheet following removal of mud with a pressurized water jet. The as-received PC sheet exhibited an absorption spectrum typical of PC glass^18^. The absorption bands, corresponding to C–H bond stretching vibration, were observed at 2874–2969 cm^−1^. The absorption band at 860–680 cm^−1^ corresponded to the bending vibration of the C–H bond, and the band at 1496 cm^−1^ was attributed to the C–H bending vibrations of methylene groups. Characteristic absorption bands of aromatic C–H bending vibration were observed at 860–680 cm^−1^ and those of aromatic C = C bending vibration were observed at 1700–1500 cm^−1^. In addition, the absorption peak at 1770 cm^−1^, which is typically associated with the C = O stretching vibration band of ethers, was observed. The FTIR spectrum of the PC sheet after mud removal displayed a peak in the range of 3070–3580 cm^−1^ that corresponded to hydroxyl groups (–OH) due to the formation of bisphenol A carbonate monomers or other substituted phenols. The presence of Si–O bonds was evidenced by the 777 and 1077 cm^−1^ peaks, corresponding to the stretching associated with SiO_2_^19^, which is remains as a residue on the surface after mud removal. In addition, the peaks at 867 and 1431 cm^−1^ were likely caused by the stretching vibrations of CO_3_^−2 ^20, which are related to calcite residue that remains after mud removal from the surface.

**Figure 10:**
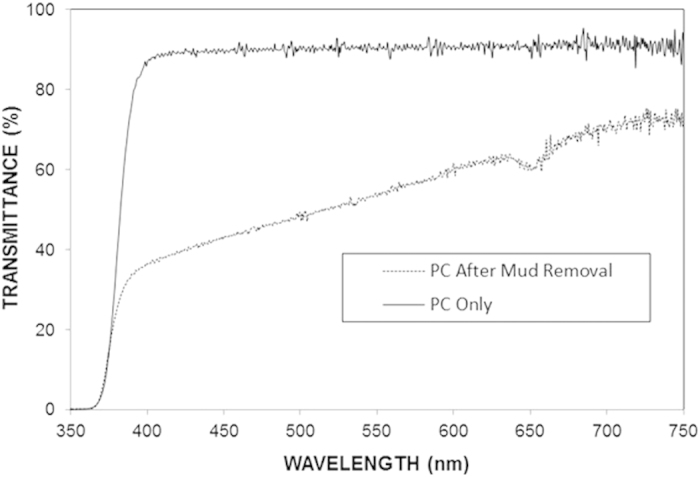
UV–visible transmittance spectra of the as-received PC sheet and the PC sheet following removal of the dry mud.

Figure 10 shows the data obtained from the UV–visible transmittance measurements of the as-received PC sheet and PC sheets following removal of mud. As observed, the transmittance decreased by nearly 45% on average for PC surface following mud removal. This reduction in the transmittance was attributed to (i) the presence of mud residues, which block incident light at the surface and (ii) molecular changes that occur at the surface of PC due to the alkaline and alkaline earth metal hydroxides attacks.

**Figure 11:**
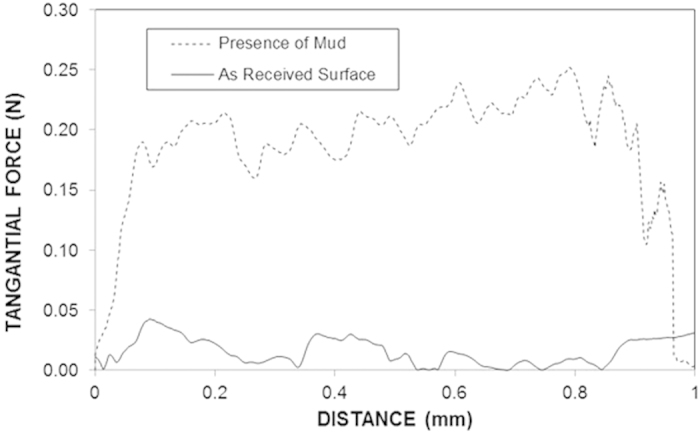
Tangential force due to friction on the as-received PC surface and on the PC surface containing mud.

Figure 11 shows the variation in the tangential force along the distance at the PC surface with and without dry mud present. The tangential force was recorded using a microtribometer during the tests for the PC surface with the dried mud and the frictional force variation on the as-received PC surface. The area under the tangential force profile describes the adhesion and frictional work required to remove the dry mud from the surface, and the area under the frictional force describes the frictional work performed^21^. Therefore, subtraction of the frictional work from the adhesion work yields the adhesion work required to remove the dry mud from the PC surface. The tangential force measurements were repeated five times, and the estimated experimental error was approximately 6%.

#### Effect of Dried Solutions

**Figure 12:**
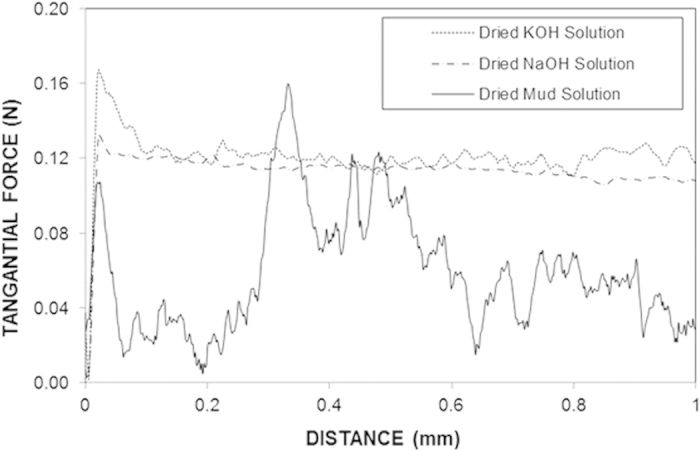
Variation in tangential force as a function of distance due to the presence of dried KOH, NaOH, and mud solutions on the PC surface.

To assess the effect of the dried solution between the dry mud and the PC surface, the liquid solution extracted from the dust was dispensed on the as-received PC surface. The adhesion force measurements were repeated for the dried mud solution only at the PC surface. Because the liquid solution extracted from the dust contained dissolved alkaline and alkaline earth metal hydroxides, the effects of the individual NaOH and KOH solutions on the adhesion force were also tested. In these tests, 10% solutions of NaOH and KOH were individually dispensed onto the PC surface, and the adhesion tests were performed after the solutions had dried. Figure 12 shows the tangential force versus the distance along the sample, as obtained from the microtribometer data for the dried solutions. The findings show that the tangential force remained high for the KOH and NaOH dried solutions. In contrast, the tangential force due to the dried mud solution was lower than that of the NaOH and KOH dried solutions. This result can be attributed to (i) the concentration of KOH and NaOH in the solution extracted from the mud prior to drying, which was less than that of the individually dispensed NaOH and KOH solutions at the PC surface and (ii) the dried mud solution containing other elements such as Fe, Cl, and Mg (Table 2). Therefore, the combined presence of the compounds formed from these elements and from NaOH and KOH in the dried mud solution lowered the tangential force required for mud removal.

Table 4 shows the adhesion work required to remove the dry mud, dry mud solution, and dry NaOH and KOH solutions from the PC surface. The adhesion work required to remove the dry mud was higher than that required to remove the remaining dry solutions from the PC surface. The van der Waals forces and the covalent bonds that formed at the PC surface were responsible for the high adhesion work required to remove the dry mud from the PC surface. To further explore the adhesion work required for the removal of the dry mud, the surface energy of the dry mud was measured using the sessile drop technique^22^. Table 5 shows the data used for the surface energy measurements of the dry mud. The surface energy of the dry mud was 55.39 mJ/m^2^ (Table 6), which was higher than that of the PC surface (i.e., 34.5 mJ/m^2^)^23^. Hence, the adhesion work required to remove the dry mud removal was high due to the high surface energy of the dry mud and presence of the dry mud solution at the interface, in which case the formation of hydroxyl groups (−OH) (Fig. 8) enhanced chemical bonding between the dry mud and the PC surface, thereby considerably enhancing the adhesion work.

**Figure 13:**
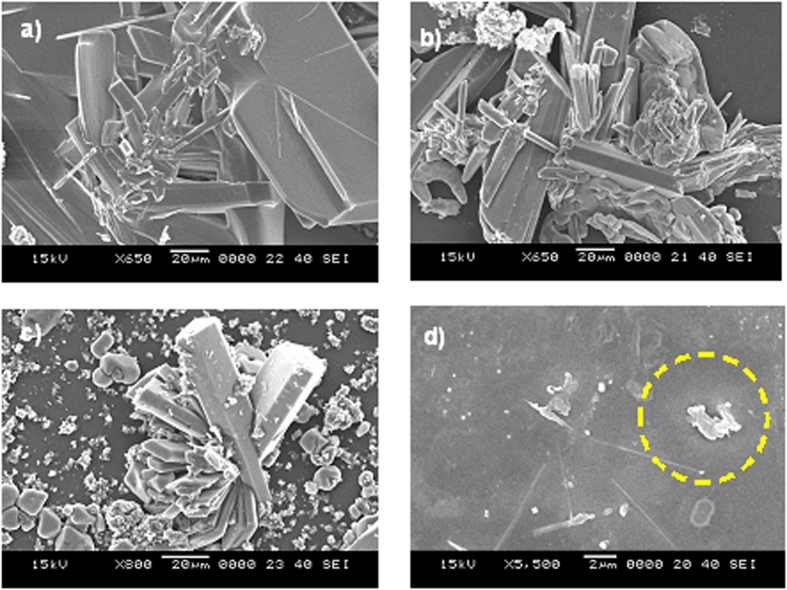
SEM images of the dried solution crystals on the PC surface: (**a**) dried NaOH solution; (**b**) dried KOH solution; (**c**) dried mud solution; and (**d**) dried CaCO_3_ solution (calcite residues and PC crystals are indicated by the circle).

**Figure 14:**
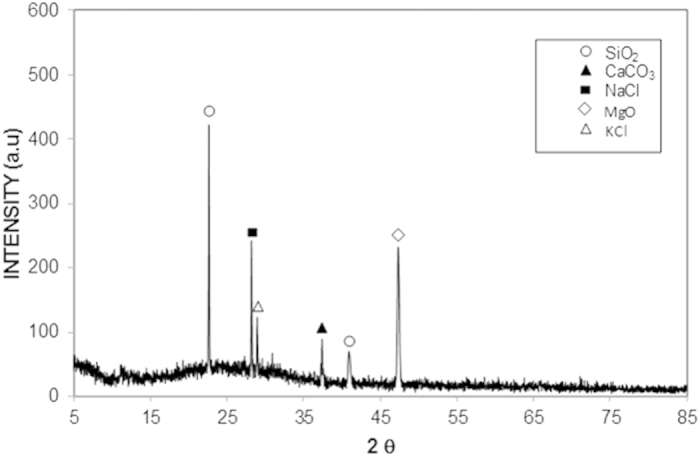
X-ray diffractogram of the PC surface after removal of mud.

Figure 13 shows the SEM images of the crystals formed at the PC surface due to the dried NaOH, KOH, and mud solutions. Close examination of the images revealed that fine crystals formed on the surface (Fig. 13a–d), which can be associated with the crystallization of the dried solution at the PC surface. Because calcium was present in the dried mud solution (Table 2) at the interface of the dry mud and the PC surface, calcite crystals formed at the PC surface, as shown in the X-ray diffractogram of the PC surface after mud removal in Fig. 14. The peak corresponding to calcite (CaCO_3_) in the diffractogram indicates the presence of calcite crystals at the PC surface. The SEM images of the crystals showed that calcite was most likely connected to polycrystalline spherulites at the PC surface, which thus contributed to the bonding between the mud and the PC surface and, in turn, increased the adhesion work. However, the presence of the NaOH and KOH solutions prior to drying resulted in shallow depth of etching which produced sub-micrometer-sized cavities at the surface, particularly in regions near the fine crystals (Fig. 7a,b). This phenomenon was more pronounced for the KOH dried solution. Local etching increased the surface roughness of the PC sheet, as shown in the line profile in Fig. 7b. The microhardness data presented in Table 3 showed that the PC after dry mud removal achieved the highest microhardness among the tested samples, followed by the dried mud solution, dried NaOH solution, and dried KOH solution. Increase in microhardness is typically associated with (i) the presence of mud residuals, such as calcite and silica, at the PC surface and (ii) chemical modification of the PC surface by the mud solution during NaOH and KOH attack. The microhardness data were obtained for the dried solutions individually dispensed onto the PC surface.

## Conclusion

Environmental dust characterization and the physical and chemical effects of mud formed from deposited dust particles on bisphenol-A PC sheets were examined in this study. The dust particles varied in size; the average particle size was 1.2 μm. Their elemental composition was non-uniform within the variously sized dust particles. Small dust particles attached to the large particles due to the forces exerted by charges present on the different materials. The aspect ratio and shape factor of the dust particles changed with particle size; however, no simple correlation was found among the dust particles, shape factor, and aspect ratio. The shape factor approached unity for small particles (≤2 μm), whereas for large particles (≥10 μm), the median shape factor approached 3. The liquid solution extracted from the dust particles contained ionic compounds (OH^−^), which increased the solution pH (pH = 8.4). When mud formed from the dust particles on the PC sheet, the dissolved alkaline and alkaline earth metal hydroxyls (OH^−^) sediments precipitated at the interface of the mud and PC surface, forming a layer in this region after drying. Alkaline hydroxyls attacked the PC surface, thereby altering the vibrational state of the macromolecules at the surface region, and increasing the microhardness and lowering the UV–visible transmittance of the resulting PC surface. In addition, the bonding of calcite and the formation of hydroxyls compounds at the PC surface increased the adhesion of the dry mud on the surface. The adhesion work required to remove the dry mud from the PC surface increased significantly because of the presence of the dried mud solution at the interface of the dry mud and the PC sheet surface. The influence of chemo-mechanical behaviour of the mud formed from the dust particles on polycarbonate surfaces is novel and significantly alters the properties of the polycarbonate. The findings provide broad insight into the performance of the polycarbonate for solar energy systems when subjected to the environmental dust and mud.

## Additional Information

**How to cite this article**: Yilbas, B. S. *et al.* Characterization of Environmental Dust in the Dammam Area and Mud After-Effects on Bisphenol-A Polycarbonate Sheets. *Sci. Rep.* **6**, 24308; doi: 10.1038/srep24308 (2016).

## Figures and Tables

**Table 1:** Elemental composition of dust (wt.%) determined by energy dispersive spectroscopy (EDS).

Si	Ca	Na	S	Mg	K	Fe	Cl	O
12.3	8.2	3.6	2.4	2.6	1.2	1.1	0.9	Balance

**Table 2:** Elemental composition of the dried mud solution on the PC surface assessed by EDS.

Dried Solution at Interface	Ca	Na	S	Mg	K	Fe	Cl	O
Spectrum 1	7.2	0.4	2.2	1.7	0.2	0.4	0.1	Balance
Spectrum 2	6.3	3.8	1.4	2.7	1.1	0.5	–	Balance
Spectrum 3	7.1	2.1	1.1	1.8	1.2	0.4	0.6	Balance
Spectrum 4	5.2	1.3	0.9	1.2	0.9	0.4	0.3	Balance
Spectrum 5	4.5	2.6	0.7	3.1	1.2	0.3	0.2	Balance
Spectrum 6	6.3	0.9	1.2	2.5	1.1	0.1	0.2	Balance
Spectrum 7	6.8	2.4	1.1	1.9	1.2	0.4	0.3	Balance

Each spectrum corresponds to a different location on the surface.

**Table 3:** Microhardness of the as-received PC sheet, dust particles, dry mud surface, PC surface after mud removal, and dried mud, NaOH, and KOH solutions.

	Hardness (HV)
As-Received PC Surface	8.2 ± 1
Dust Particle (size ≥10 μm)	26.3 ± 1
Dry Mud Surface	20.2 ± 1
PC Surface after Mud Removal	15.1 ± 1
Dried Mud Solution	13.6 ± 1
Dried NaOH Solution	11.7 ± 1
Dried KOH Solution	10.4 ± 1

The errors are based on several measurements.

**Table 4:** Measured adhesion work required for the removal of dry mud, dried mud solution, dried NaOH solution, and dried KOH solution from the PC surface.

	Adhesion work (mJ)
Dry Mud	0.15558
Dried Mud Solution	0.03664
Dried NaOH Solution	0.08396
Dried KOH Solution	0.09167

The friction of the as-received PC surface was 0.000603 mJ.

**Table 5:** Lifshitz–van der Waals components and electron–donor parameters used in the simulation^22^.

	*γ*_*L*_ (mJ/m^2^)	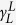 (mJ/m^2^)	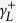 (mJ/m^2^)	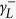 (mJ/m^2^)
Water	72.8	21.8	25.5	25.5
Glycerol	64	34	3.92	57.4
Ethylene Glycol	48	19	0.41	1.28

L indicates the liquid phase; *γ*_*L*_ is the liquid surface tension; *γ*^*L*^ is the a polar component due to the Lifshitz–van der Waals intermolecular interactions; and *γ*^+^ and *γ*^−^are the electron acceptor and electron donor parameters, respectively, of the acid–base component of the solid and liquid surface free energy.

**Table 6:** Lifshitz–van der Waals components and electron–donor parameters of the laser-treated smooth surface.

*γ*_*S*_ (mJ/m^2^)	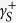 (mJ/m^2^)	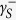 (mJ/m^2^)	*γ*^*P*^ (mJ/m^2^)	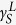 (mJ/m^2^)
55.39	0.2688	48.17	7.197	48.19

S indicates the solid phase; *γ*^+^ and *γ*^−^ are the electron acceptor and electron donor parameters, respectively, of the acid–base component of the solid and liquid surface free energy; *γ*^*P*^ is due to electron–acceptor and electron donor intermolecular interactions; and 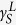 is the interfacial solid–liquid free energy.
